# Polydopamine-Cu(II) Ions Functional Coatings on Zinc Wire: Surface Characterization and Degradation Behavior

**DOI:** 10.1021/acsmaterialsau.6c00093

**Published:** 2026-05-20

**Authors:** Md Tanvir Hossain, Hamid Reza Bakhsheshi-Rad, Bashir Ahamed, Erico Freitas, Bruce P. Lee, Jeremy Goldman, Jaroslaw W. Drelich

**Affiliations:** † Department of Materials Science and Engineering, 3968 Michigan Technological University, Houghton, Michigan 49931, United States; ‡ Department of Materials Engineering Na.C., Islamic Azad University, Najafabad, Iran; § Department of Biomedical Engineering, Michigan Technological University, Houghton, Michigan 49931, United States

**Keywords:** biodegradable implant, zinc, polydopamine coating, degradation behavior, stent application

## Abstract

Bioactive surface modification of degradable zinc (Zn) vascular implants is essential for controlling biodegradation and supporting vascular healing. Here, we describe the fabrication of polydopamine coating (PDA-Cu) containing up to ∼0.8 wt % Cu^2+^ ions on Zn substrates using an immersion-assisted mussel-inspired polymerization strategy. The coating is composed of densely packed PDA-Cu nanospheres with diameters of ∼130–170 nm, exhibiting an even distribution of Cu ions within the PDA matrix via Cu^2+^-catechol coordination. Thirty-day leaching tests confirmed a steady release of 0.01–0.04 ppm of Cu ions per 5 days from Zn/PDA-Cu samples in Hank’s balanced salt solution (HBSS). *In vitro* nitric oxide (NO) assay demonstrated enhanced catalytic NO generation in 1 mM SNAP (*S*-nitroso-*N*-acetylpenicillamine) solution. Additionally, the PDA-Cu coating reduced the Zn corrosion rate from ∼0.8 mm/year to ∼0.4 mm/year and increased its corrosion resistance in HBSS.

## Graphical abstract

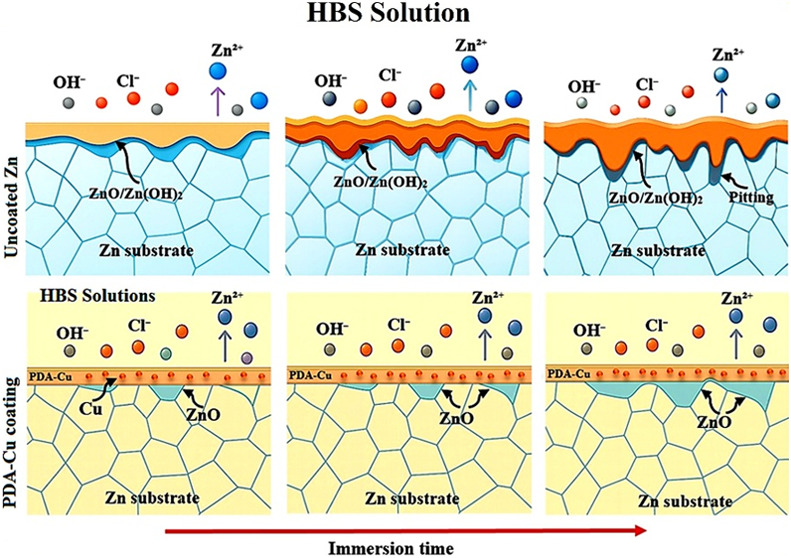

## 1 Introduction

Cardiovascular disease remains the leading cause of morbidity and mortality worldwide, with coronary artery disease being a major contributor.^1^ The use of vascular stents has significantly improved clinical outcomes by restoring blood flow in blocked vessels. Among biodegradable metals, Zn has emerged as a promising candidate.^2–4^ It offers a moderate corrosion rate, suitable mechanical strength, and physiological relevance as an essential trace element involved in many biological functions.^5–7^ However, bare Zn surfaces present limitations, including nonuniform corrosion and poor interaction with vascular cells.^8,9^ These issues can hinder endothelialization and promote inflammation. Therefore, surface modification strategies, particularly those using organic or hybrid coatings, have been explored to enhance the interface between the stent material and the vascular environment.^10–12^

Polydopamine (PDA), a bioinspired polymer derived from dopamine self-polymerization, offers a versatile platform for surface functionalization due to its strong adhesion to a wide range of substrates, chemical reactivity, and biocompatibility.^13^ Importantly, PDA can chelate transition metal ions, such as copper (Cu^2+^), which catalyze the decomposition of endogenous S-nitrosothiols (RSNOs) to release NO under physiological conditions.^14^ PDA-Cu systems have demonstrated endothelium-mimicking behavior when applied to various substrates, including transition metal ions and polymers, showing improved NO release, reduced thrombogenicity, and enhanced endothelial cell proliferation.^15,16^

For biodegradable Zn-based vascular stents, precise control of the corrosion rate, particularly during the early stage of implantation, is critical for maintaining structural stability and ensuring predictable degradation behavior.^2^ Although Zn exhibits a moderate overall corrosion rate, its initial degradation can be localized and nonuniform, leading to surface instability and accelerated material loss. Such behavior can compromise the integrity and uniformity of the stent before the desired degradation timeline is achieved.^8^ Therefore, the development of surface coatings that act as effective barriers to regulate corrosion kinetics, suppress localized attack, and promote uniform degradation is essential for improving the reliability and performance of Zn-based biodegradable stent materials.

Recent studies have demonstrated that PDA-Cu coatings enhance the biofunctionality of vascular implants by enabling sustained NO generation, supporting endothelialization, and reducing the incidence of thrombosis and restenosis. PDA-Cu assemblies on 316L stainless steel (SS) stents have exhibited these multifunctional effects in situ.^17^ In addition, copper-incorporated PDA coatings on magnesium (Mg) alloys have improved corrosion resistance and influenced immune and vascular cell responses.^18^ The use of PDA-Cu coatings on Zn-based biodegradable substrates has not been thoroughly investigated. Limited data exist regarding the effects of these coatings on Zn surface characteristics, corrosion behavior, and biological interactions.^19^ Addressing this gap is important because Zn-based materials offer moderate degradation rates and biocompatibility for vascular applications.^8^ Controlled corrosion is necessary because rapid degradation can compromise mechanical integrity. Furthermore, the chemical analysis of the coating, surface morphology, catalytic activity in NO generation, and ion-release profiles of PDA-Cu coatings on Zn require systematic evaluation to design improved stent materials.

This report describes the fabrication of functional PDA nanosphere coatings on biodegradable zinc vascular substrates. The PDA-Cu coating improves substrate corrosion resistance by blocking Zn ion release from the implant material and fluid movement to the implant.

## 2 Experimental Section

### 2.1 Materials and Coating Preparation

Zn wire (>99.99%; diameter = 0.25 mm) as drawn and disk-shaped Zn substrates purchased from Goodfellow (England) were used for coating. The disk-shaped samples’ had a diameter of 6 mm and a thickness of 5 mm were then polished with 400, 600, 800, and 1200-grade grit SiC paper to enhance smoothness and uniformity, ensuring optimal surface conditions for subsequent coating procedures. The samples were then ultrasonically cleaned in deionized (DI) water for 5 min and subsequently dried using compressed air. Polydopamine (PDA) coatings were prepared by dissolving dopamine hydrochloride (*M*_W_ = 189.64 g/mol, Thermo Scientific, USA) at a concentration of 0.25 g/L in 10 mM Tris-HCl buffer (pH 8.5). For the PDA-Cu coatings, copper­(II) chloride (CuCl_2_) was added to the dopamine solution at different concentrations of 5, 10, and 20 mg/L, as illustrated in Figure 1. These concentrations were selected to investigate the effect of Cu incorporation while remaining far below the solubility limit of CuCl_2_ (757 g/L at 25 °C), ensuring solution homogeneity and minimizing cytotoxic risk associated with excess Cu ions.

**Figure 1:**
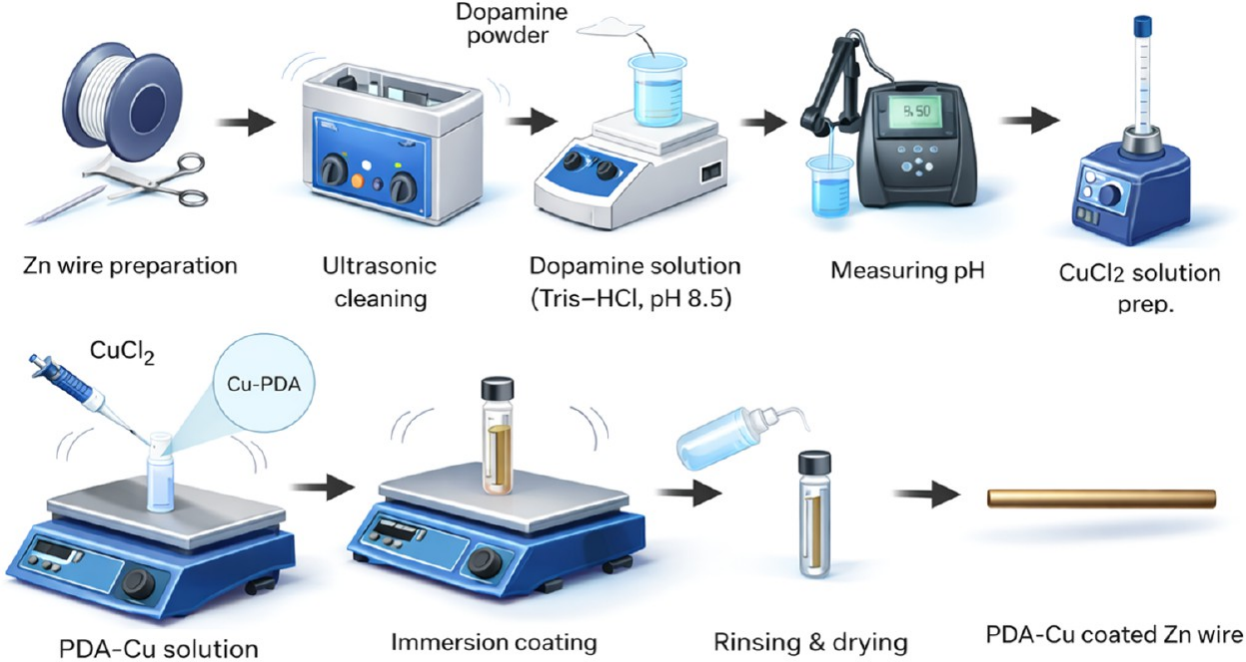
Schematic illustration of the synthesis and PDA-Cu coating process.

The Zn samples were immersed in the prepared PDA or PDA-Cu solutions and maintained under gentle stirring at room temperature (pH 8.5) for 8 h to allow mussel-inspired oxidative polymerization of dopamine. In this process, it is hypothesized that Cu^2+^ ions form coordination complexes with the catechol and amine functional groups of PDA. This interaction is expected to promote metal–polymer cross-linking and enhance the adhesion of the film (Figure 1). After coating, the samples were systematically removed and rinsed with ethanol to remove unbound residues, then air-dried under ambient conditions. The specimens were designated as bare Zn (control), PDA, PDA-Cu5, PDA-Cu10, and PDA-Cu20 for subsequent characterization and analysis presented in Supporting Information (Figure S1).

### 2.2 Coating Surface Characterization

The microstructural analysis of bare Zn substrates, PDA, PDA-Cu5, PDA-Cu10, PDA-Cu20 coated Zn was examined using a Thermo Fisher Apreo 2 FE-SEM operated in high-vacuum mode at 20 kV accelerating voltage, 1.6 nA beam current, and 10 mm working distance. SEM images were collected with the through-lens (in-lens, T2) secondary electron detector, which also gets some backscatter electrons. Samples were mounted on aluminum stubs using conductive carbon adhesive tabs and gently blown with nitrogen (air) to remove loose particulates. Representative fields were recorded over nominal magnifications ∼250× to 2,000×. Representative micrographs were acquired at nominal magnifications ranging from 250× to 2,000×. Elemental mapping was conducted using an Oxford Instruments AZtec UltimMax EDS system integrated with the FE-SEM (image size: 1024 pixels; dwell time: 10 μs; working distance: 10 mm). Particle size distribution of PDA nanospheres was determined from SEM images using ImageJ (NIH, USA). A total of 160 particles (*N* = 160) were measured across multiple regions, and their diameters were used to generate a histogram, with the mean and standard deviation reported. Moreover, cross-sectional specimens of the coated Zn wires were prepared using a Leica Ultracut UCT ultramicrotome equipped with a diamond knife to produce smooth and uniform sections. The prepared cross sections were subsequently analyzed by SEM to determine coating thickness and assess interfacial integrity. Coating thickness was determined from SEM cross-sectional images using ImageJ software. A total of 90 measurements (*N* = 90) were taken from different regions of the coating to ensure statistical reliability. The thickness values were analyzed and reported as a mean value and standard deviation.

Scanning transmission electron microscopy (STEM) analysis was employed to evaluate the coating morphology and elemental distribution. PDA films with varying copper ion concentrations were prepared by drop casting dopamine-Cu precursor solutions onto the ultrathin (<5 nm thick) lacey carbon-coated gold (Au)-TEM grids (300 mesh). The grids were then left at ambient conditions for 24 h to allow complete solvent evaporation. Samples were handled with care to prevent contamination or mechanical damage and stored in a clean, dust-free environment until analysis. TEM samples were mounted on a FEI (low X-ray background) double-tilt holder. To minimize contamination during imaging, a beam-shower technique was applied before STEM observation to stabilize surface adsorbates via low-dose electron pre-exposure.

STEM analysis was performed using an aberration-corrected FEI Titan Themis 200 kV STEM equipped with spherical aberration correction. High-angle annular dark-field scanning transmission electron microscopy (HAADF-STEM) was used to visualize the spatial distribution of copper within the PDA matrix at atomic resolution. The camera length was set to 110 mm to allow a collection angle on the HAADF detector of 70–200 mrad, optimizing for composition contrast. Energy-dispersive X-ray spectroscopy (EDS) was conducted in both spectral and mapping modes to confirm the elemental composition and assess the uniformity of copper incorporation.

### 2.3 Coating Chemistry Characterizations

The UV–vis absorption spectra were recorded using a Shimadzu UV-1800 spectrophotometer to observe changes in peak intensity and position over time, indicating the progression of PDA polymerization and the formation of PDA-Cu^2+^ complexes. Samples were placed in a 1 cm path-length quartz cuvette, and spectra were collected over the wavelength range 300 to 1100 nm. A Tris buffer was used as a blank for baseline correction.

Attenuated Total Reflectance Fourier Transform Infrared (ATR-FTIR) spectra were acquired on a Jasco FT/IR-4600 spectrometer (JASCO, Japan) equipped with a diamond single-reflection ATR accessory (45°). Spectra were recorded from 4000 to 500 cm^–1^ at a resolution of 2 cm^–1^ by coadding 50 scans for each sample and 50 background scans (clean diamond crystal) to identify the coating chemical bonding and the change after incorporation of Cu. PDA and Cu-incorporated PDA coatings were analyzed directly on their surfaces under constant applied contact pressure without further sample preparation. This technique is well-suited for detecting characteristic polymeric functional groups, such as O–H or N–H stretching and C=C bonding.

Additionally, X-ray photoelectron spectroscopy (XPS) was performed using a PHI 5800 spectrometer (Physical Electronics, USA) with a monochromatic Al Kα source (*h*ν = 1486.6 eV, 15 kV, 250 W; ∼400 μm spot) to investigate the bonding of PDA-Cu-coated samples. The analysis chamber base pressure was 5 × 10^–9^ mbar. Survey spectra (0–1200 eV) were acquired at a pass energy of 117.4 eV (0.8 eV step), and high-resolution C 1s, N 1s, O 1s, and Cu 2p spectra were collected at 23.5 eV pass energy with 0.05 eV steps, averaging 10 sweeps. All spectra were processed and analyzed using CasaXPS software. Prior to peak fitting, binding energies were calibrated by referencing the C–C/C–H peak to ∼284.9 eV. A Shirley background was applied to all spectra. Peak deconvolution was performed using mixed Gaussian–Lorentzian (GL) line shapes, with peak positions assigned based on literature-reported binding energies for corresponding chemical states. During fitting, appropriate constraints were applied to ensure physically meaningful results. The full width at half-maximum (FWHM) was allowed to vary within a narrow, consistent range for similar components to maintain fitting consistency and reliability. Elemental compositions were quantified using relative sensitivity factors (RSFs) provided by the instrument database.

### 2.4 *In Vitro* Leaching of Cu Ions

The release of Cu^2+^ from the coated samples was quantified using inductively coupled plasma optical emission spectroscopy (ICP-OES, PerkinElmer Optima 7000DV). Samples with a diameter of 0.25 mm and a length of 4 mm were immersed in 10 mL of Hank’s balanced salt solution (HBSS) at 37 °C in a thermostatically controlled incubator, following the immersion protocol of ASTM G31–21. The solution volume and sample-to-solution ratio were kept constant to ensure comparability across all test groups. Aliquots of the immersion medium were collected at predetermined time points (e.g., 5, 10, 15, 20, 25, and 30 days). To maintain sink conditions and avoid concentration saturation, an equal volume of fresh HBSS was replenished after each sampling. The collected solutions were filtered using 0.22 μm syringe filters to remove any suspended particles before ICP-OES measurement. ICP-OES was calibrated using certified Cu standards, and the measurements were conducted in triplicate (*n* = 3) for each time point to ensure statistical accuracy.

### 2.5 *In Vitro* Nitric Oxide Generation Assay

Nitric oxide (NO) release from the coated wires was quantified using a Sievers 280i nitric oxide analyzer (GE Analytical Instruments, Chicago, IL, USA), based on chemiluminescence detection. Bare Zn and PDA-Cu coated wires with varying Cu^2+^ concentrations were tested and compared against control samples. All samples were pre-equilibrated prior to NO measurement. For NO donor activation, *S*-nitroso-*N*-acetylpenicillamine (SNAP) was used as the physiological NO source. Wires (∼0.25 mm diameter, 4 mm length) were suspended in 2 mL of 1 mM SNAP solution prepared in Chelex 100-treated phosphate-buffered saline (PBS). Chelex treatment was performed by dissolving 1 PBS tablet in 200 mL deionized (DI) water, followed by the addition of 10 g of Chelex 100 resin to remove trace metal ions that could interfere with NO detection. The solution was stirred for at least 1 h and then filtered prior to use.

A 10 mL SNAP stock solution (11.92 mg SNAP in 5 mL treated PBS) was prepared and diluted to obtain final working concentrations. Samples were incubated in the SNAP solution for 2 h at room temperature. During this period, NO release was continuously monitored in real time, with data recorded every second. The headspace above the reaction vessel was purged with high-purity nitrogen to carry the evolved NO gas into the analyzer. All NO generation experiments were performed in triplicate (*n* = 3) for statistical validity.

### 2.6 Electrochemical Corrosion Characterizations

Electrochemical corrosion testing was conducted in HBSS whose pH was adjusted to 7.4 using 1 M HCl and 1 M NaOH solutions. Measurements were performed in a conventional three-electrode cell employing a platinum counter electrode, a saturated calomel reference electrode (SCE), and the prepared sample as the working electrode. Potentiodynamic polarization scans were acquired at 37 ± 1 °C under naturally aerated conditions at a scan rate of 0.166 mV s^–1^ over a 1.0 V potential window using VersaStudio software for control and data acquisition. This protocol provided a basis for comparative evaluation of the corrosion behavior, degradation progression, and surface uniformity of coated and uncoated specimens under physiologically relevant conditions. The corrosion rate (CR) of the samples was calculated from the relationship between corrosion rate and corrosion current density according to ASTM G59–97 as follows

1 $$\mathrm{CR}\left(\frac{\mathrm{mm}}{\mathrm{year}}\right)=3.27\times 10^{-3}\frac{i_{\mathrm{corr}}E.W.}{\rho }$$

where *i*_corr_ is the current density (μA/cm), ρ is the density of the substrate (g/cm^3^), and E.W. is the equivalent weight of the substrate, calculated as follows

2 $$E.W.=\frac{1}{\sum \frac{n_{i}f_{i}}{w_{i}}}$$

where *f*, *w*, and *n* are mass fraction, atomic weight, and valence of the elements in the substrate. Corrosion rates are reported as mean values with standard deviations from three experiments for each coated and uncoated sample. To ensure reliability, all experiments were conducted three times. According to electrochemical findings, the polarization resistance (*R*_p_) of a coating can be determined via the following formula:^20^

3 $$R_{p}=\frac{\beta_{a}\times \beta_{c}}{2.303\left(\beta_{a}+\beta_{c}\right)i_{\mathrm{corr}}}$$

where *R*_p_ is the polarization resistance, β_a_ and β_c_ are the anodic and cathodic Tafel constants, respectively, and *i*_corr_ is the corrosion current density. The corroded samples were further examined to evaluate changes in coating morphology, chemical phase development, and surface chemistry using FE-SEM, and FTIR analyses. The corroded surfaces were gently scratched with a clean razor blade, and FTIR was subsequently used to characterize the resulting scratches.

## 3 Results and Discussion

### 3.1 Microstructural Characterization

The surface morphologies and elemental distributions of the bare and coated Zn wires are presented in Figure 2. The uncoated Zn wire exhibits a relatively smooth surface with faint polishing marks, indicating the absence of organic contaminants or thick oxide films (Figure 2a).

**Figure 2:**
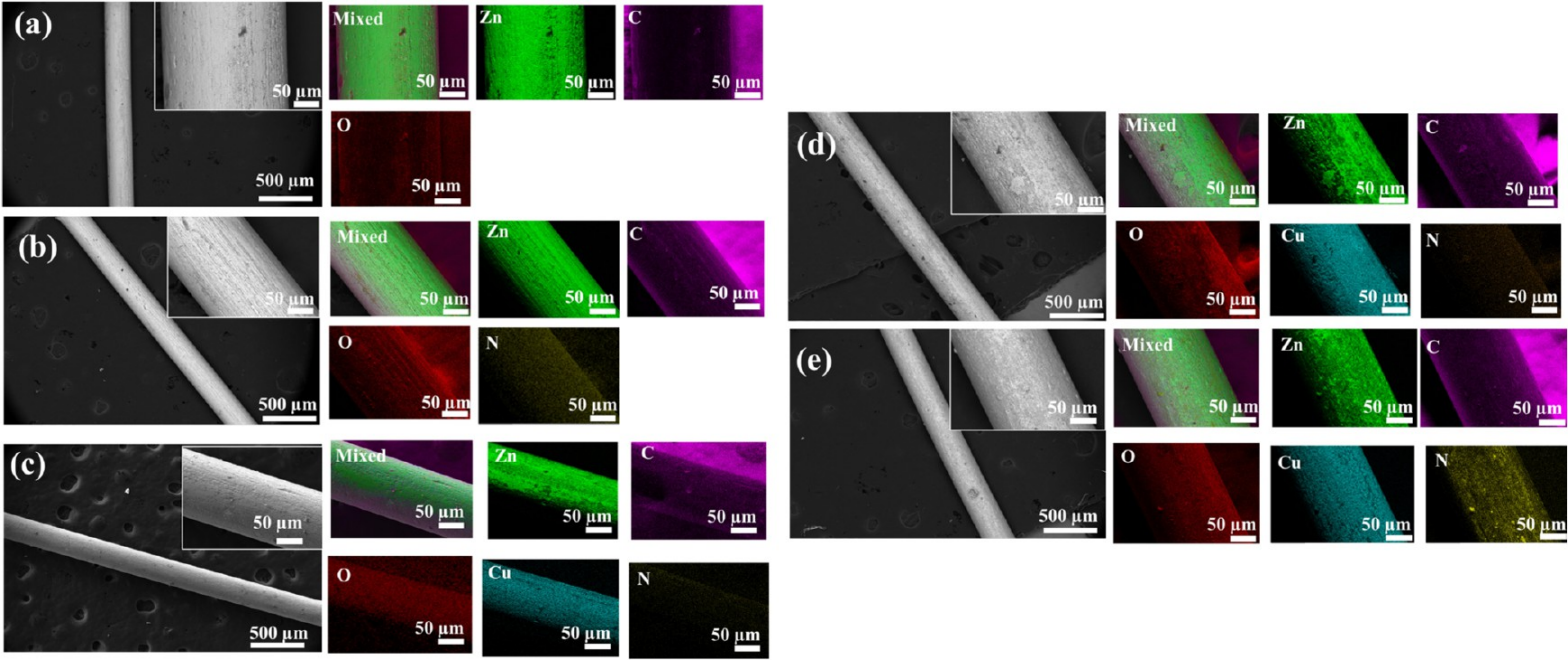
Secondary electrons SEM images showing the surface morphology of (a) bare Zn wire, (b) PDA-coated Zn wire, (c) PDA-Cu5, (d) PDA-Cu10, and (e) PDA–Cu20 coated Zn wire, along with corresponding EDS mapping images illustrating elemental distribution.

The EDS elemental mapping reveals a prominent Zn signal, accompanied by minor oxygen. The minimal carbon signal further underscores the cleanliness of the metallic surface before coating. Following PDA deposition, the surface exhibits a slight increase in roughness and a more uniform texture, indicating the formation of a continuous organic coating layer (Figure 2b). The EDS maps reveal strong carbon and oxygen signals along with a distinct nitrogen distribution, characteristic of PDA.

Incorporation of Cu into the PDA layer modifies the surface characteristics. For the PDA-Cu5 and PDA-Cu10-coated Zn wire, the morphology shows fine granular features, indicating the development of a metal–polymer coating (Figure 2c,d). EDS elemental mapping demonstrates a homogeneous distribution of C, O, N, and Cu, confirming the distribution of Cu within the PDA matrix. When the Cu concentration reached 20 mg/L, the surface displayed microaggregated nodules. This change in morphology is attributed to enhanced PDA cross-linking and partial agglomeration of Cu-catechol complexes at higher metal ion concentrations (Figure 2e). The corresponding EDS maps exhibit intense signals from C, O, N, and Cu, with only a minor contribution from Zn. The reduced Zn signal is consistent with the presence of a surface coating; however, this observation may also be influenced by coating thickness, the electron interaction volume, and signal attenuation effects, and therefore does not definitively confirm complete substrate coverage. The observed localized inhomogeneities may result from uneven polymerization caused by excess Cu content. To avoid excessive aggregation of PDA-Cu nanospheres, Cu concentration above 20 mg/L was not pursued.

To further substantiate these morphological observations at higher resolution, high-magnification SEM imaging of the PDA-Cu coating (Figure 3a) reveals a uniform distribution of spherical nanoparticles with diameters of 150 ± 20 nm. These nanospheres form a multilayered structure (approximately 4–5 layers) across the surface. TEM analysis at higher magnification (Figure 3b) further corroborates the coating’s spherical morphology. Such nanostructures are characteristic of PDA-based systems formed via oxidative self-polymerization of dopamine in the presence of Cu ions. This observation is consistent with previous studies; for instance, Wu et al.^21^ reported the formation of spherical polydopamine nanostructures. Cross-sectional SEM analysis (Figure 3c) confirms the formation of a continuous and conformal coating on the Zn wire surface. The coating exhibits thickness of 700 ± 180 nm, determined from approximately 90 measurements taken at different locations along the cross-section. This thickness corresponds to the deposition of roughly 4–5 layers of PDA nanospheres, resulting in effective surface coverage.

**Figure 3:**
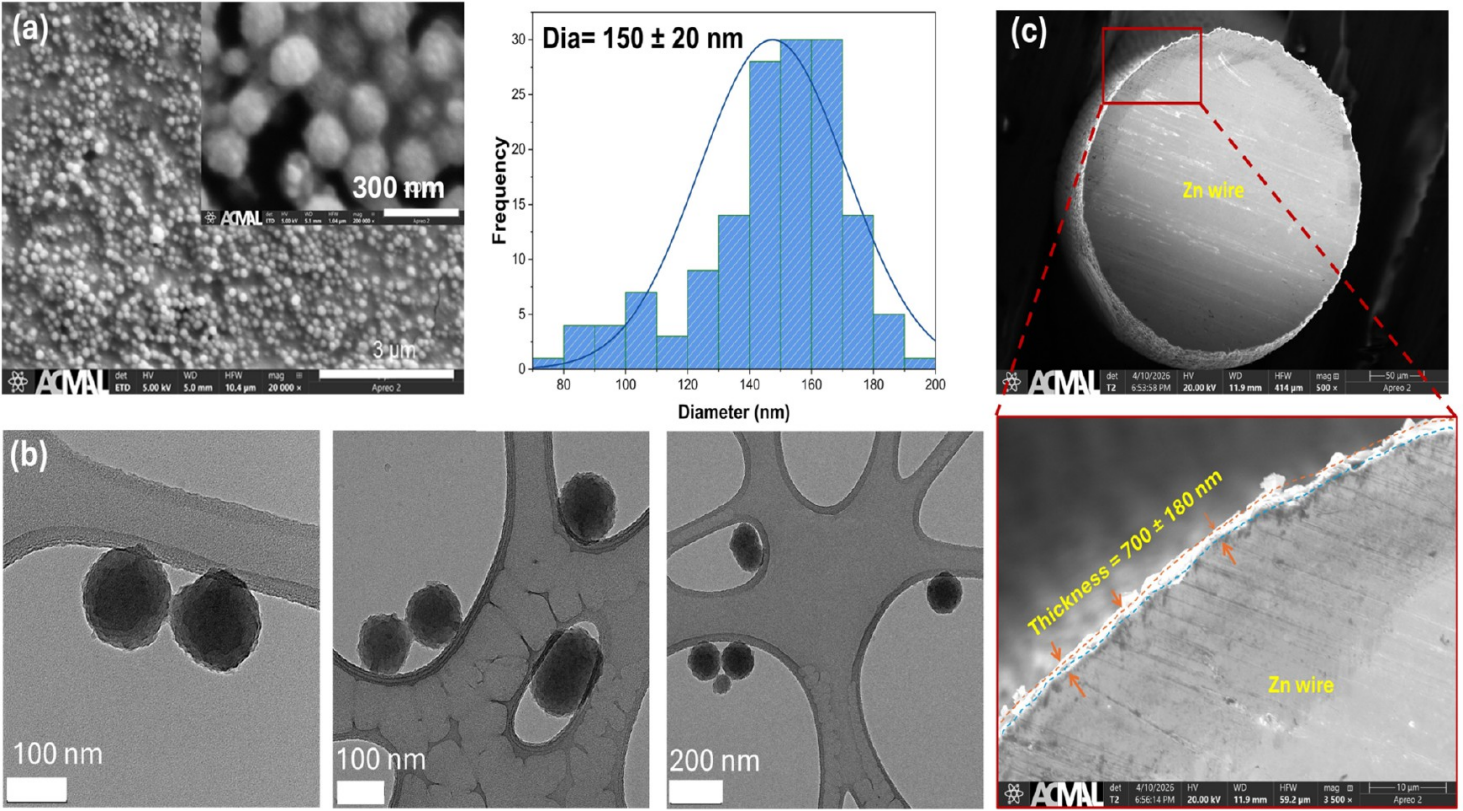
Surface morphology and particle size distribution of the PDA-Cu coating on Zn substrates, (a) SEM image showing the formation of uniformly distributed PDA-Cu spherical nanoparticles, with diameters of 150 ± 20 nm, as confirmed by size distribution analysis, (b) TEM micrographs (prepared on lacey carbon-supported gold TEM grids) at different magnifications, (c) SEM image showing coating thickness from the cross-section of the Zn wire.

Furthermore, TEM analysis enabled high-resolution visualization of spherical PDA particles, providing insights into constituent elements and distributions (Figure 4). In all samples, carbon remains the main element, confirming the dominance of the PDA matrix. Copper peaks appear in the PDA-Cu coatings, indicating successful metal incorporation, while oxygen and nitrogen show broader peaks due to varied bonding states within the polymer.^22,23^ A minor chlorine signal likely originates from residual CuCl_2_ used during the coating process. The PDA elemental map without Cu appears less defined. When Cu is introduced, the PDA map becomes clearer and more intense. This improvement occurs because Cu ions accelerate dopamine polymerization and promote faster deposition of nanospheres on the surface, resulting in a denser, more continuous PDA-Cu layer.

**Figure 4:**
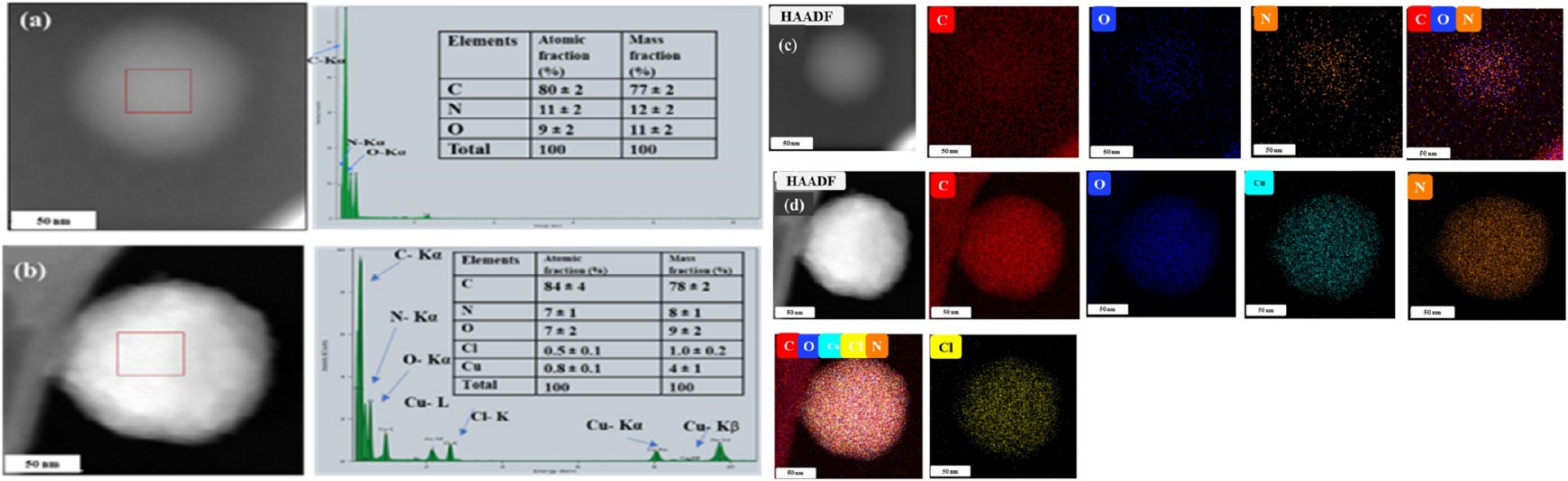
STEM and elemental analysis of PDA and PDA-Cu20 coatings. (a, b) STEM images and corresponding EDS spectra with elemental compositions. (c, d) HAADF-STEM images with corresponding EDS elemental maps (C, O, N, Cu, and Cl) showing the spatial distribution of elements within the coatings (scale bar = 50 nm).

The TEM images of the PDA coating (Figure 4a) display a nearly spherical particle with a smooth, homogeneous contrast, indicating a dense, amorphous organic structure. The corresponding EDS spectrum reveals only C (80 ± 2 atom %), N (11 ± 2 atom %), and O (9 ± 2 atom %), consistent with the catecholamine-derived polymer matrix. Upon Cu incorporation at 20 mg/L (Figure 4b), the TEM image reveals a spherical shape. EDS confirms the presence of Cu (0.8 ± 0.1 atom %) and Cl (0.5 ± 0.1 atom %), in addition to C (84 ± 4 atom %), N (7 ± 1 atom %), and O (7 ± 2 atom %). The emergence of Cu peaks at ∼0.9 keV (Cu–Lα) and ∼8.0 keV (Cu–Kα) reflects successful doping of Cu within the PDA framework. It should be noted that EDS signal intensity reflects relative elemental presence and does not directly quantify the exact coating composition.

The distribution of elements and the localization of copper are illustrated in Figure 4c,d. Elemental mapping supports the observed compositional trends. In the pristine PDA sample (Figure 4c), the uniform spatial distribution of carbon, oxygen, and nitrogen confirms the chemical homogeneity of the amorphous polymer, with no detectable metallic species. In contrast, the PDA-Cu20 coating (Figure 4d) exhibits a distinct spherical domain in the HAADF image. The homogeneous overlap of C, O, N, and Cu signals indicates a uniform dispersion of copper within the polymerized PDA nanosphere.^24,25^ Overall, SEM and TEM analyses confirm the formation of PDA and PDA-Cu coating layers on the Zn wire surface, indicating uniform surface coverage. EDS spectra and elemental maps confirm the presence of constituent polymer elements and the incorporation of Cu, indicating their homogeneous distribution throughout the coating.

### 3.2 Coating Chemistry

The UV–vis spectra of polydopamine (PDA), both in the absence and presence of Cu^2+^ ions, illustrate notable optical and mechanistic differences (Figure 5(a,b)). In the absence of Cu^2+^, the UV–vis spectrum of PDA displays a minor absorption peak at ∼420 nm, indicative of uncoordinated PDA. This peak primarily results from π → π* transitions, associated with the aromatic catechol or quinone moieties. A time-dependent increase in absorbance suggests progressive polymerization of dopamine into PDA, leading to the formation of conjugated structures such as cross-linked indole, catechol, and quinone systems. In contrast, the PDA-Cu^2+^ complex displays two broad absorption bands at ∼465 nm and ∼700–800 nm. Notably, the original PDA peak shifts from ∼420 nm to ∼460–465 nm, indicating coordination between Cu^2+^ and the PDA matrix.^26^ The absorption band located around ∼345 nm is likely associated with ligand-to-metal charge transfer (LMCT) from the oxygen atoms of catechol to Cu^2+^ ions.^27^ In contrast, the broad absorption observed in the range of 700–800 nm can be attributed to d-to-d transitions of Cu^2+^ within a distorted octahedral coordination environment.^28^

**Figure 5:**
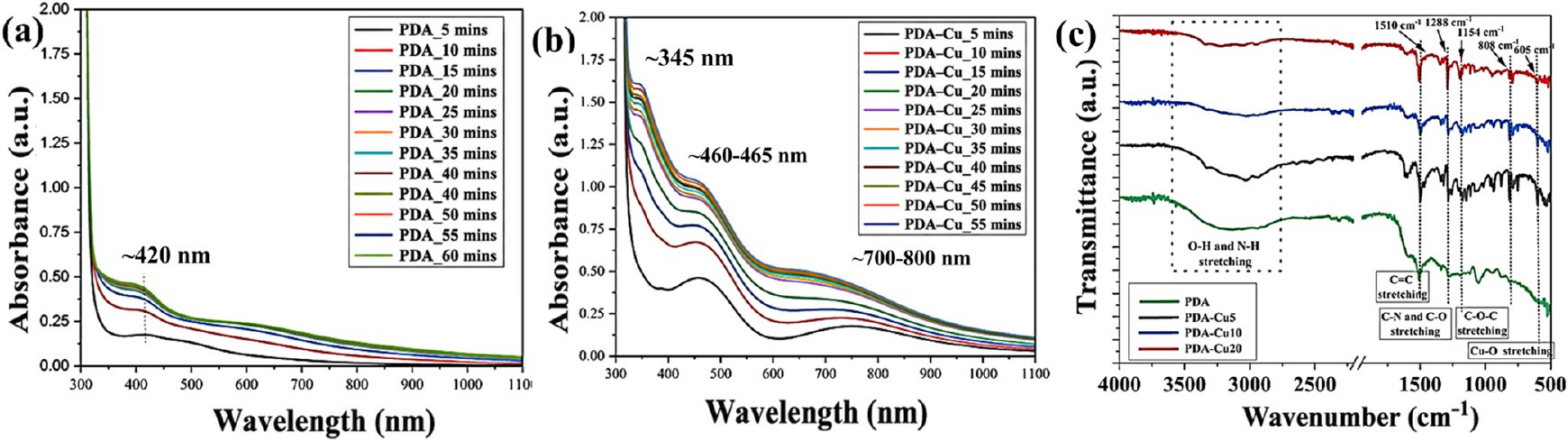
UV–vis spectra of (a) PDA solution and (b) 20 mg/L Cu^2+^ solution; (c) FTIR spectra of PDA and PDA coated with different concentrations of Cu.

The enhanced absorption intensity in PDA-Cu^2+^ suggests that Cu^2+^ coordination stabilizes PDA, increasing conjugation and electronic delocalization. Mechanistically, Cu^2+^ binds to PDA through catechol −O– groups and secondary amines formed during polymerization, adopting a distorted octahedral geometry.^29^ Additionally, Cu^2+^ accelerates dopamine polymerization via redox cycling (Cu^2+^ to Cu^+^), generating radicals that enhance cross-linking.^30^ These findings highlight the catalytic and structural advantages of Cu^2+^-coordinated PDA, making it suitable for coating applications.

FTIR spectroscopy was employed to investigate the functional groups present in the PDA coating and to assess its interaction with copper ions (Cu^2+^) at varying concentrations. Figure 5(c) displays the FTIR spectra of PDA and Cu^2+^-incorporated PDA coatings (PDA-Cu10, and PDA–Cu20), collected in the wavenumber range of 400–4000 cm^–1^. The PDA spectrum exhibits a broad absorption band in the region of 3200–3500 cm^–1^, attributed to overlapping O–H and N–H stretching vibrations, characteristic of phenolic and amine groups, respectively. These groups are integral to the adhesion and chelating properties of PDA.^31^ A sharp band observed at 1510 cm^–1^ corresponds to aromatic C=C stretching, indicating the presence of the indole or catechol ring structures formed during dopamine oxidative polymerization. The peaks at 1288 cm^–1^ and 1154 cm^–1^ are assigned to C–N stretching and C–O stretching vibrations, respectively, while the signal at 808 cm^–1^ is associated with C–O–C symmetric stretching, which is consistent with previous studies on PDA coatings.^32^

Upon incorporation of Cu^2+^, distinct spectral changes were observed. The broad O–H/N–H stretching band showed slight broadening and intensity reduction, suggesting partial coordination of copper ions with hydroxyl and amine groups. The intensity of the C–N and C–O stretching bands decreased with increasing Cu^2+^ concentration, indicating the involvement of these functional groups in metal ion binding.^33^ Most notably, a new absorption band emerged around 605 cm^–1^, attributed to Cu–O stretching, confirming successful coordination between Cu^2+^ ions and PDA functional groups. This Cu–O vibration became more pronounced at higher copper concentrations, especially in PDA-Cu20.

These observations are in agreement with previous reports on metal-doped PDA systems. For example, Li et al.^18^ reported similar spectral shifts and the appearance of a Cu–O band in copper-loaded PDA films, indicating chelation via catechol and amine groups. Likewise, Lee et al.^34^ demonstrated that transition metal ions such as Cu^2+^ and Fe^3+^ form coordination complexes with PDA through bidentate binding, evidenced by changes in the O–H and C–N stretching regions of the FTIR spectrum. Overall, the FTIR data confirm the successful binding of Cu^2+^ ions to PDA, involving coordination through catechol (O–H) and amine (N–H) moieties. The analysis of Cu–O stretching bands reveals a strong correlation with the concentration of Cu^2+^ ions, indicating efficient and adjustable metal incorporation within the PDA matrix. A more comprehensive examination of these findings is detailed in the XPS results.

XPS was conducted to examine the surface chemistry, elemental states, and coordination interactions present in both the PDA and PDA-Cu coatings. The survey spectrum indicates the presence of peaks for C 1s, N 1s, and O 1s across all samples, highlighting the organic characteristics of polydopamine (Figure 6a). In contrast, the distinctive Cu 2p_3/2_ and Cu 2p_1/2_ peaks are observed only in the PDA-Cu5, PDA-Cu10, and PDA-Cu20 coatings, confirming the successful immobilization of copper ions via dip-coating. The intensity of the Cu signals correlates with the concentration of Cu^2+^, indicating a greater loading of copper in PDA-Cu20 compared to PDA-Cu10 and PDA-Cu5. As anticipated, no Cu-related peaks were observed in the pristine PDA coating. The high-resolution C 1s spectra (Figure 6d–g) for all coatings were deconvoluted into four primary components: C–C/C–H (∼284.9 eV), C–N or C–O (∼286.2 eV), C = O (∼288.5 eV), and a π–π* satellite (∼290.7 eV), which is typical for aromatic structures.^18^ The presence of C–N and C–O peaks confirms the formation of the catecholamine-derived PDA backbone. Notably, the relative fraction of carbonyl (C=O) increases in the PDA-Cu coatings compared to pristine PDA, suggesting partial oxidation of catechol to quinone groups during Cu^2+^ coordination. This shift supports the proposed chelation mechanism, where Cu^2+^ ions preferentially bind to oxidized catechol (quinone) and amine groups.

**Figure 6:**
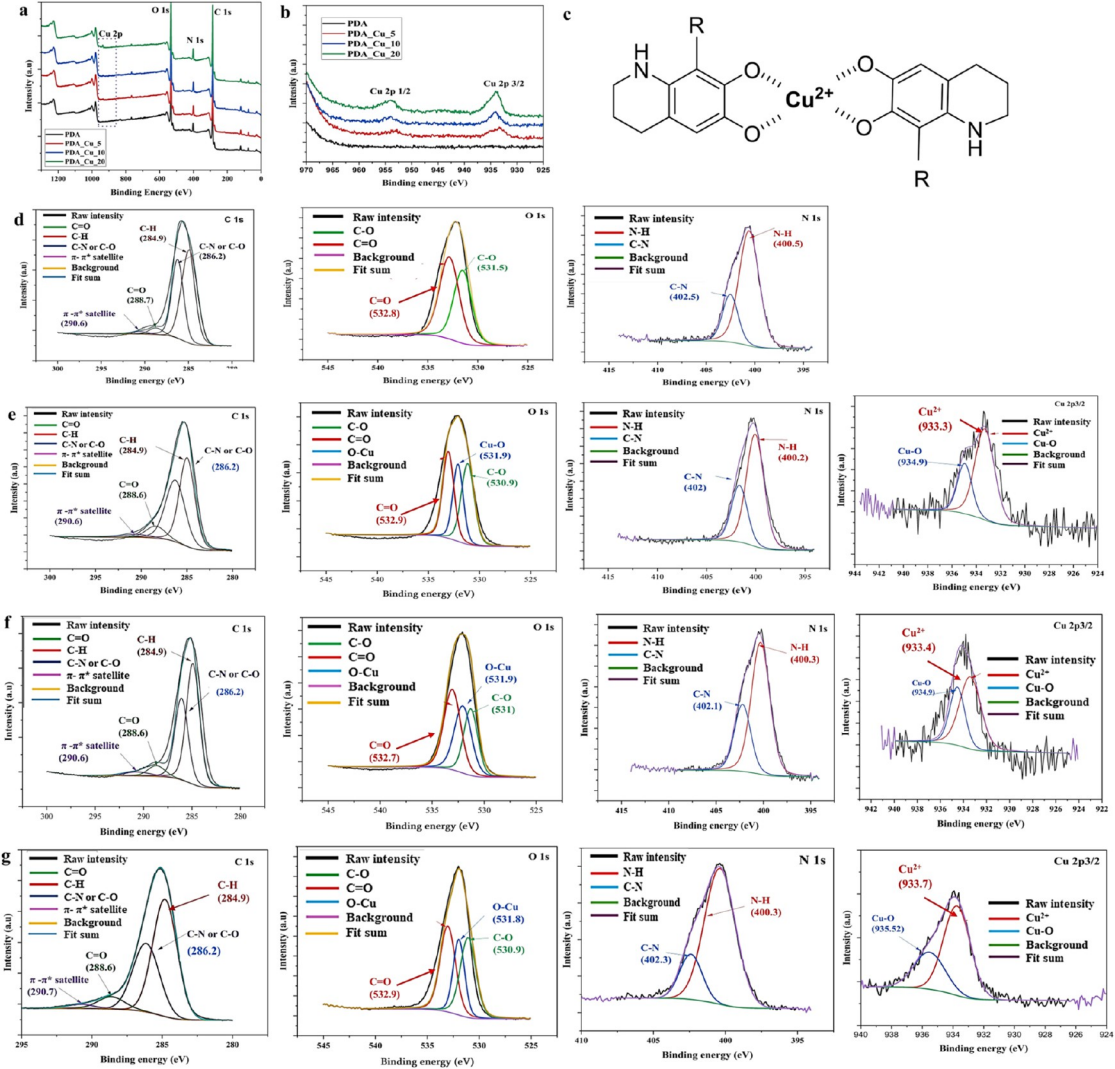
XPS analysis of the PDA and PDA-Cu coatings: (a) survey spectra of PDA, PDA-Cu10, and PDA-Cu20 samples; (b) high-resolution Cu 2p spectra for the Cu-containing coatings; (c) proposed chemical structure/coordination mechanism of the PDA-Cu complex; high-resolution spectra of C 1s, O 1s, N 1s, and Cu 2p for (d) PDA coating, (e) PDA-Cu5 (f) PDA-Cu10 coating, and (g) PDA-Cu20 coating.

As shown in the case of N 1s and evidence of Cu–N interaction, the N 1s spectra (Figure 6d–g) exhibit two major peaks centered at ∼400.1 eV (assigned to amine/secondary amine N–H) and ∼402.1 eV (attributed to protonated or imine-type nitrogen, C=N).^19^ PDA-Cu coatings show a slight shift and increased intensity in the higher binding energy component (∼402 eV), indicating nitrogen coordination or electron withdrawal due to Cu^2+^ binding. This observation aligns with the expected coordination between Cu^2+^ and the amine/indole groups of PDA.

Moreover, the O 1s spectrum of PDA primarily consists of C–O (532.9 eV) and C=O (531.5 eV) components. Upon Cu incorporation (Figure 6e–g), an additional peak emerges at ∼530.9 eV, corresponding to Cu–O bonding.^35^ This peak confirms the formation of Cu-catechol complexes. Concurrently, the C–O intensity slightly decreases relative to C=O, further indicating oxidation of hydroxyl groups to quinones during metal coordination.

The high-resolution Cu 2p_3/2_ spectra (Figure 6e–g) show dominant peaks at ∼933.7 eV with a weak satellite structure at ∼941–943 eV, consistent with Cu^2+^ species rather than Cu^0^ or Cu^+^. A secondary feature at ∼935.5 eV is attributed to Cu–O chelation within the PDA matrix.^18^ The absence of metallic Cu peaks indicates that copper is immobilized in an oxidized and coordinated state rather than as metallic clusters or nanoparticles. Based on these observations, a schematic of the proposed PDA-Cu coordination is shown in Figure 6c. Cu^2+^ ions form chelation complexes with both catechol oxygen (Cu–O) and amine or imine nitrogen (Cu–N) groups, leading to a stable organic-metal structure. The increase in carbonyl and imine content, along with the emergence of Cu–O peaks, further validates this mechanism.

**Figure 7:**
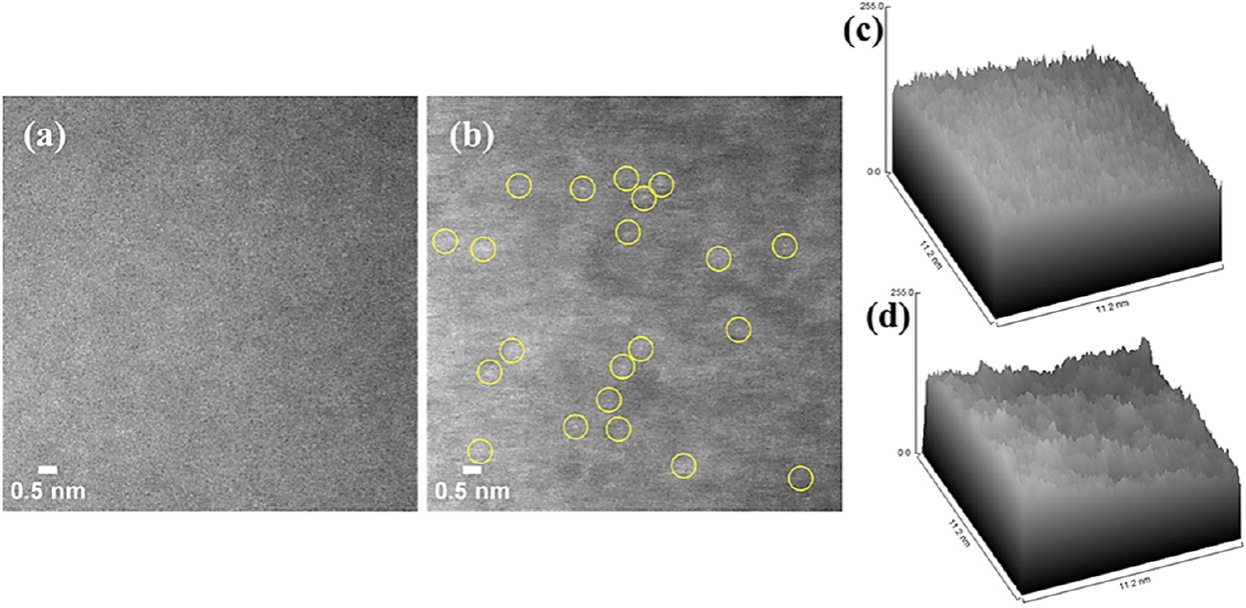
Aberration-corrected HAADF-STEM investigation of PDA and PDA-Cu coatings. (a, b) High-resolution STEM images of PDA, PDA-Cu20 (c, d) 3D surface plot in the form of the PDA and PDA-Cu20 coating specimens.

Collectively, these observations support the proposed coordination mechanism between PDA and Cu, as illustrated in Figure 6c. Cu^2+^ ions coordinate simultaneously with both the catechol oxygen (Cu–O) and amine groups, leading to the formation of a stable metal–polymer network. The increased presence of carbonyl and imine groups, along with the detection of Cu–O bonding signatures, further substantiates the role of Cu in facilitating dopamine oxidation and the development of a durable PDA-Cu composite coating.

Figure 7 displays the aberration-corrected HAADF-STEM analysis of both PDA and PDA-Cu coatings, integrating atomic-resolution imaging, three-dimensional surface plot. The high-resolution aberration-corrected STEM images presented in Figure 7(a,b) illustrate a distinct structural evolution with the introduction of Cu into the PDA matrix. The PDA film (Figure 7a) shows a consistent amorphous contrast that is characteristic of an organic polymer made up of light elements (C, N, O), without any noticeable high-*Z* scattering centers. However, with the incorporation of Cu at a concentration of 20 mg/L (Figure 7b), discrete bright spots emerge, indicating the presence of localized high-*Z* Cu centers within the PDA network. The bright contrast observed in the high-angle annular dark-field (HAADF) images results from incoherent Rutherford scattering, where the signal intensity significantly increases with atomic number (*Z*).^36^ Consequently, heavier elements such as Cu (*Z* = 29) appear as bright spots, whereas lighter elements like C, N, and O from the PDA matrix produce much weaker contrast.^37^ These isolated bright features, therefore, further confirm that Cu atoms are molecularly chelated within the polymer network through coordination with catechol groups. 3D surface plot of coating specimens confirm the PDA-20Cu coating exhibits rougher surface morphology than that of PDA coatings (Figure 7c,d).

### 3.3 *In Vitro* Cu Ion Leaching and NO Generation

Detection of Cu^2+^ ion release is important to ensure controlled delivery and to avoid potential cytotoxic effects associated with elevated copper levels. Figure 8 illustrates the *in vitro* release behavior of Cu^2+^ ions from PDA-Cu coatings with different initial Cu loadings (5, 10, and 20 mg/L) immersed in simulated body fluid (SBF). As shown in Figure 8a, all PDA-Cu coatings exhibit a time-dependent Cu^2+^ release pattern characterized by a mild initial burst followed by a steady-state release phase. However, PDA-only coating exhibited no detectable Cu^2+^ release throughout the 30-day immersion period. The daily Cu^2+^ concentration remains below 0.05 ppm for both samples, indicating a low leaching level. The magnitude of Cu^2+^ release correlates positively with the initial Cu incorporation within the PDA matrix, where PDA-Cu20 shows the highest daily release, followed by PDA-Cu10 and 5. Importantly, there is a decreasing release of Cu ions from the PDA-Cu5 sample, which leads to a depleting amount of Cu ions in the coating.

**Figure 8:**
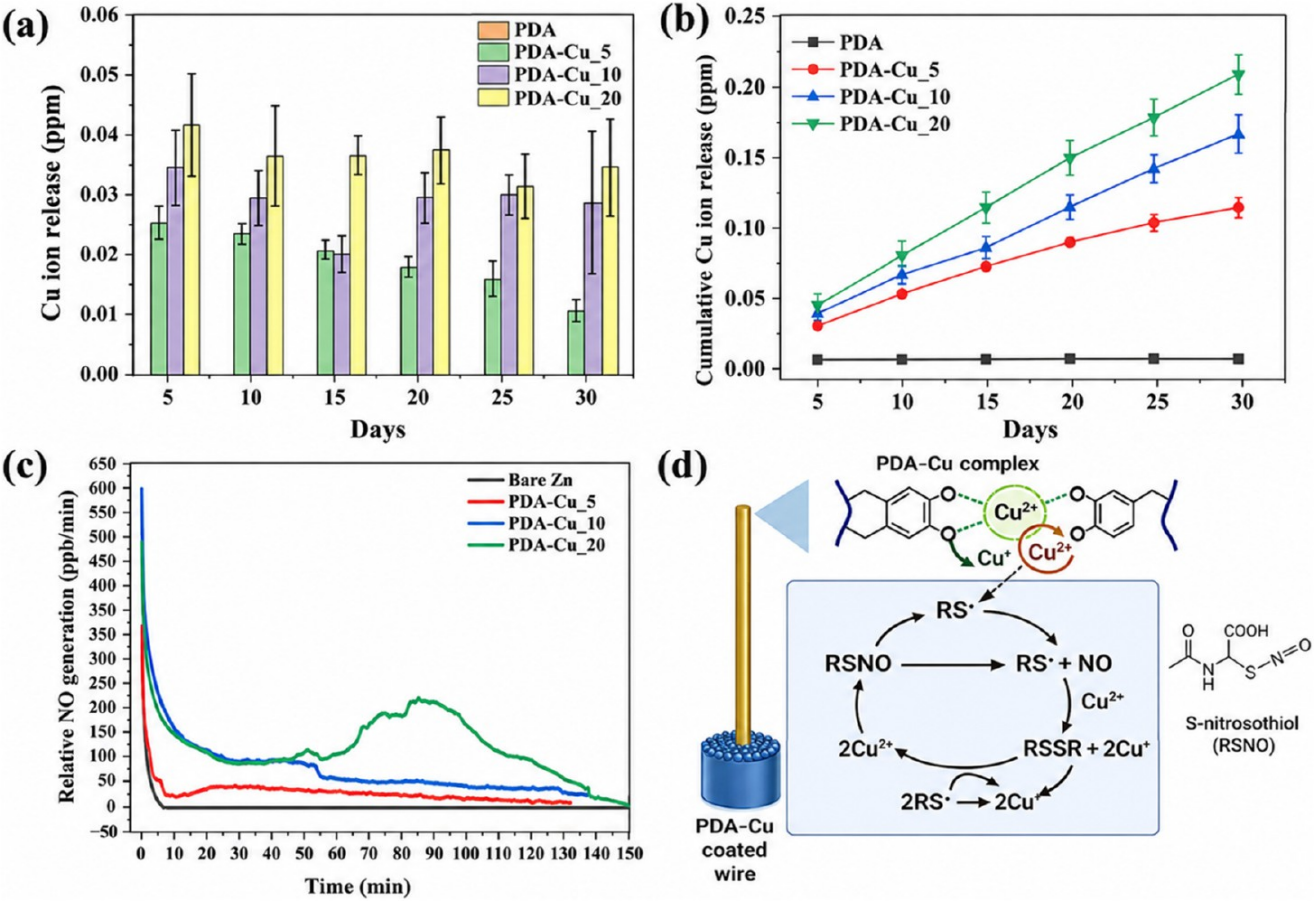
*In vitro* Cu ion release from PDA-Cu^2+^ coating (5, 10, 20 mg/L Cu ion concentration) in SBF (a) Cu ion release over days, (b) cumulative Cu ion release, and (c) relative nitric oxide (NO) generation profiles of bare Zn wire and PDA-Cu-coated Zn with varying Cu^2+^ concentrations in SNAP-containing physiological solution (pH 7.4), (d) reaction associated with NO generation from the coated surface in the presence of Cu^2+^ via RSNO decomposition.

The corresponding cumulative release profile (Figure 8b) demonstrates a nearly linear increase in Cu^2+^ concentration over 30 days, suggesting that the release process is governed by diffusion through the hydrated PDA network and gradual chelation, dissociation of Cu-catecholate complexes. Such linear kinetics are indicative of a controlled and sustained release mechanism, in contrast to uncontrolled burst leaching often observed in physically adsorbed systems. The steady elution rate implies that Cu^2+^ ions are progressively liberated from coordination sites within the PDA framework without structural collapse, preserving the coating’s integrity throughout immersion. The higher cumulative release from PDA-Cu20 reflects a greater population of Cu-ligand coordination sites available for exchange, whereas PDA-Cu5 maintains the lowest overall release, consistent with its lower initial Cu content and denser cross-linked structure.

At the end of the 30-day test, the cumulative leaching of Cu^2+^ from Cu5, Cu10, and Cu20 reached moderate concentrations of 0.11 ± 0.01 ppm, 0.17 ± 0.01 ppm, and 0.22 ± 0.01 ppm, respectively. Notably, these concentrations of released Cu^2+^ ensuring adequate availability of Cu^2+^ for catalytic NO generation.^38^ Notably, the Cu10 and Cu20 exhibited nearly identical Cu^2+^ release kinetics profiles, which is in agreement with the aforementioned XPS results of similar Cu atomic ratios in the as-prepared coatings. Overall, the PDA-Cu coatings demonstrate a predictable, concentration-dependent Cu^2+^ release behavior. Such controlled release may be advantageous for long-term vascular applications; however, further biological evaluation is required to confirm cytocompatibility and assess potential ion-induced effects.

In addition, nitric oxide (NO) is an essential vasoregulatory and antithrombotic signaling molecule that maintains endothelial homeostasis, inhibits platelet adhesion, and suppresses smooth muscle cell proliferation.^39^ In physiological systems, endogenous *S*-nitrosothiols (RSNOs) such as *S*-nitrosoalbumin (SNA) and *S*-nitrosoglutathione (GSNO) act as natural NO reservoirs.^40^ Transition metal ions, particularly Cu^2+^, can catalyze the decomposition of RSNOs by cleaving the S–N bond, leading to NO release through redox cycling between Cu^2+^ and Cu^+^ states. The PDA-Cu coatings were designed to exploit this catalytic property while providing controlled ion release and biocompatibility at the implant surface. Figure 8c shows the relative NO generation rate (in PPB/s) of bare Zn and PDA-Cu-coated Zn wires with different Cu loadings (5, 10, and 20 mg/L) in SNAP-containing phosphate-buffered saline (pH 7.4). Bare Zn wire exhibits a negligible NO signal, confirming its inability to catalyze RSNO decomposition. In contrast, all PDA-Cu coatings demonstrate a distinct NO generation response, with the magnitude and duration directly correlated to the Cu content within the coating. The PDA-Cu20 sample shows the highest NO flux, peaking around 200 ppb/s, followed by Cu10 and Cu5, which produce a comparatively lower but steady signal.

All coatings exhibit an initial burst in NO generation during the early reaction phase (0–1000 s), attributed to the rapid redox activation of surface Cu^2+^ sites in the presence of RSNO donors. After this stage, the release stabilizes (Cu10, Cu20), governed by the diffusion of RSNO molecules to catalytic sites and the possible gradual reduction of Cu^2+^/Cu^+^ cycles mediated by the catechol/quinone moieties of PDA. The PDA matrix thus plays a dual role: (i) acting as a redox mediator that could facilitate cyclic Cu^2+^ to Cu^+^ transitions, and (ii) regulating ion leaching to prevent excessive Cu release and cytotoxicity.

At higher Cu loading (20 mg/L), increased NO output is observed, likely due to a greater density of catalytically active Cu sites and enhanced RSNO decomposition. Coatings containing 5–20 mg/L Cu exhibit sustained NO generation over extended durations, indicating stable catalytic activity. Such controlled NO release may be advantageous for vascular applications; however, its effects on vasodilation, thrombosis prevention, and endothelialization require further biological validation. Figure 8d schematically illustrates Cu-mediated catalytic decomposition of RSNO species at the PDA-Cu interface, leading to NO generation through Cu^2+^/Cu^+^ redox cycling. The sulfur-containing coproducts are shown only in a simplified manner; detailed sulfur radical and disulfide transformations are not intended to imply a fully resolved elementary mechanism. Further investigations are required to fully elucidate the NO generation pathways, including the identification of transient intermediates, the role of Cu oxidation states, and the influence of physiologically relevant RSNO/thiol environments.

### 3.4 Effect of Coating on Zn Substrate Corrosion

The electrochemical properties of different specimens, including PDA and PDA-Cu coatings and the uncoated sample, were investigated based on the Tafel plots shown in Figure 9a. It is well-known that corrosion resistance is usually directly related to two basic parameters, namely corrosion potential (*E*_corr_) and corrosion current density (*i*_corr_).^41^ Typically, the *i*_corr_ value is a measure for determining the corrosion rate obtained by fitting curves; the lower this value, the higher the anticorrosion property of the coating. In this study, the corrosion potential of PDA–Cu20 layers was measured to be about −1020 mV_SCE_, which was slightly higher than the value recorded for the uncoated sample (−1050 mV_SCE_). Also, the corrosion current density decreased from 81 μA/cm^2^ in the uncoated sample to 38 μA/cm^2^ in the PDA–Cu20 coating. The *i*_corr_ value of the PDA coating was also obtained to be 36 μA/cm^2^, which was slightly lower than that of the PDA-Cu20 coating (38 μA/cm^2^). The order of increasing corrosion current density for the samples was observed as follows: PDA < PDA-Cu20 < uncoated Zn (Table 1). The PDA and PDA-Cu20 coatings demonstrated a significant enhancement in corrosion resistance by increasing the corrosion potential and reducing the corrosion current density compared to bare zinc. After applying PDA and PDA-Cu20 coatings, the polarization resistance (*R*_p_) of the samples increased significantly, confirming enhanced anticorrosion behavior. Specifically, the *R*_p_ values for the PDA and PDA-Cu20 coatings were recorded at 0.75 and 1.64 kΩ·cm^2^, respectively, which is noticeably higher than the uncoated zinc surface, measured at 0.46 kΩ·cm^2^. Correspondingly, the corrosion rates for the coated samples decreased to 0.35 mm·year^–1^ for PDA and 0.37 mm·year^–1^ for PDA–Cu20, both of which are lower than those of bare zinc. These enhancements can be attributed to the formation of a protective PDA-based barrier layer, which restricts the transport of corrosive species to the zinc substrate. The findings indicate that both PDA and PDA-Cu20 coatings effectively inhibit zinc corrosion and slow down the degradation of the substrate when compared to the uncoated surface. To more accurately evaluate the protective performance of these layers, EIS tests were performed. The Nyquist and Bode plots of the bare Zn, PDA and PDA-Cu coatings after different immersion times in HBSS solution along with the equivalent circuit used are presented in Figure 9b–d. In the electrical equivalent circuits, the components *R*_s_, *R*_c_, CPE_c_, CPE_dl_ and *R*_ct_ represent the electrolyte resistance, coating layer resistance, coating pseudocapacitive capacitance, double layer capacitance and charge transfer resistance, respectively.^33,42^

**Figure 9:**
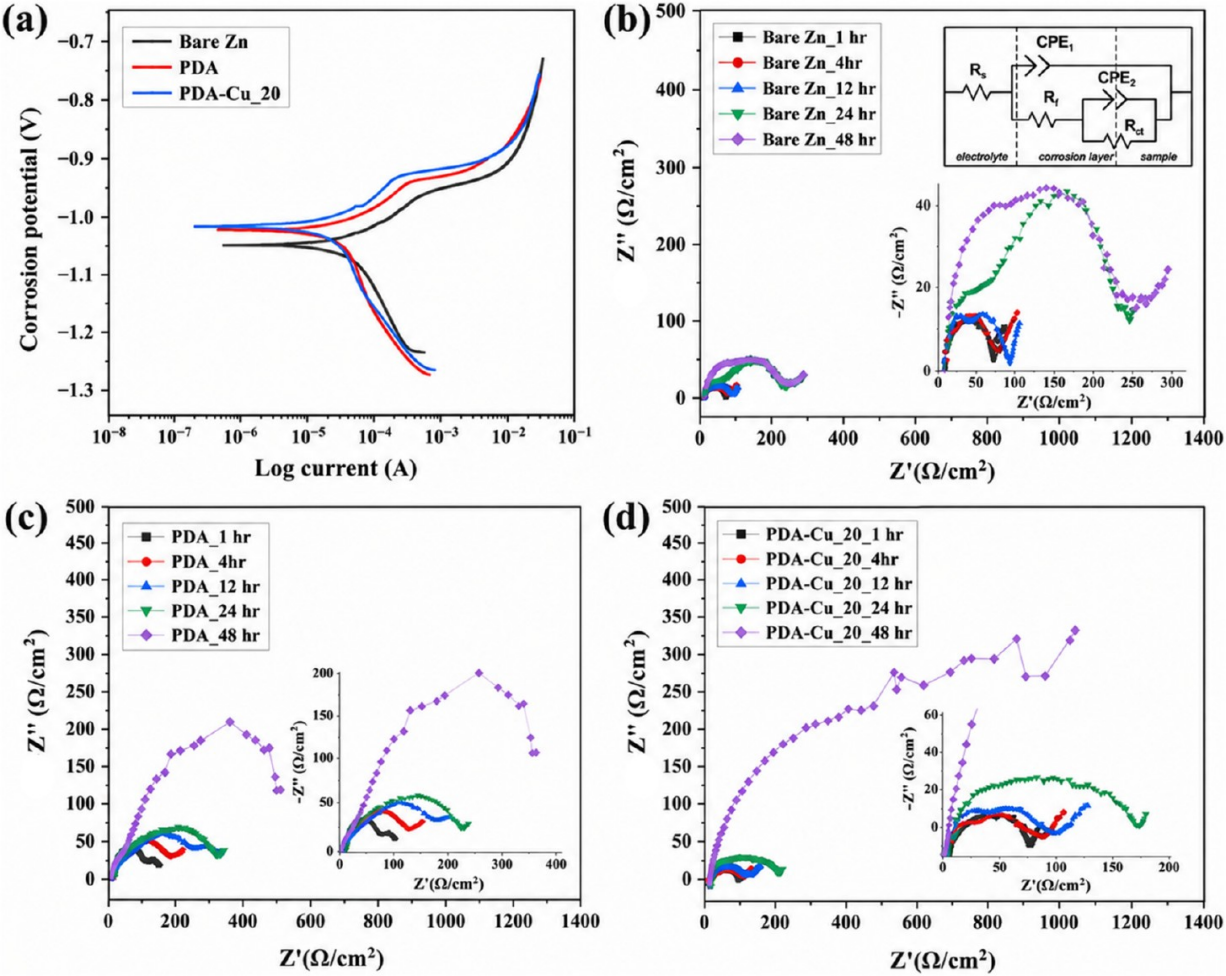
(a) Potentiodynamic polarization curves (b) equivalent electronic circuit model of bare Zn. (b–d) Nyquist plots obtained from electrochemical impedance spectroscopy (EIS) measurements for bare Zn, PDA, PDA-Cu coatings after immersion in HBSS solution for 1, 4, 12, 24, and 48 h.

**Table 1:** Summary of Electrochemical Corrosion Parameters of Coated and Uncoated Samples

Sample	Corrosion potential (mV_SCE_)	Cathodic slope, β_c_ (mV/decade)	Anodic slope, β_a_ (mV/decade)	Polarization resistance, *R*_p_ (kΩ·cm^2^)	*i*_corr_ (μA·cm^–2^)	Corrosion rate (mm/year)
Bare Zn	–1050 ± 45	513 ± 20	105 ± 8	0.46 ± 0.03	81 ± 5	0.80 ± 0.20
PDA coated	–1025 ± 35	345 ± 18	77 ± 6	0.75 ± 0.05	36 ± 3	0.35 ± 0.15
PDA-Cu20 coated	–1020 ± 40	500 ± 20	95 ± 7	0.91 ± 0.07	38 ± 4	0.37 ± 0.15

The Nyquist and Bode plots obtained at immersion times of 1, 4, 12, 24, and 48 h show the electrochemical behavior of the different specimens. Usually, an increase in the diameter of the capacitive ring in the Nyquist spectrum is an indication of an increase in corrosion resistance. It is observed that the PDA and PDA-Cu20 sample has semicircles with a larger diameter than the uncoated Zn surface at all time intervals, which indicates a higher impedance and greater resistance to the penetration of the corrosive environment during the first 24 h of contact. This finding indicates that the coated layer can effectively protect the substrate from rapid degradation. On the other hand, the uncoated surface showed only a small, and incomplete semicircle in the Nyquist plot. This semicircle corresponded to the midfrequency region (10^4^ to 10^1^ Hz) in the phase diagram and was related to the thin, unstable passive layer formed on the surface.^43,44^

Furthermore, the total impedance value was minimum at 1 h and maximum at 48 h, which is probably due to the partial formation of oxide layers on the metal surface due to localized oxidation. The PDA-Cu coating exhibited improved resistance to corrosion attack. The gradual increase in the diameter of the semicircle in the high-frequency region from 1 to 48 h of immersion indicates the stability of the coating and its ability to prevent the penetration of corrosive ions into the substrate.^44^ It was observed that the uncoated zinc surface exhibited two time constants after only 1 h of immersion, indicating rapid penetration of the corrosive solution to the interface between the surface and the substrate. In contrast, the PDA and PDA-Cu coatings showed such behavior only after 48 h, confirming that these layers act as an effective barrier against electrolyte penetration and prevent direct contact of the corrosive medium with the substrate.

Typically, the impedance modulus at the lowest frequency (*Z*_f_ = 0.01 Hz) of the Bode diagram is considered as an effective index for evaluating the barrier properties of coatings against electrolyte penetration.^43,44^ After 1 h of immersion, the uncoated Zn sample had a *Z*_f_ = 0.01 Hz value of 1.0 × 10^2^ Ω·cm^2^, while this value increased significantly to 8 × 10^2^ Ω·cm^2^ after 48 h of immersion in the solution (Figure 10a). This change can be attributed to the gradual formation of oxide layers and stable corrosion products on the surface.

**Figure 10:**
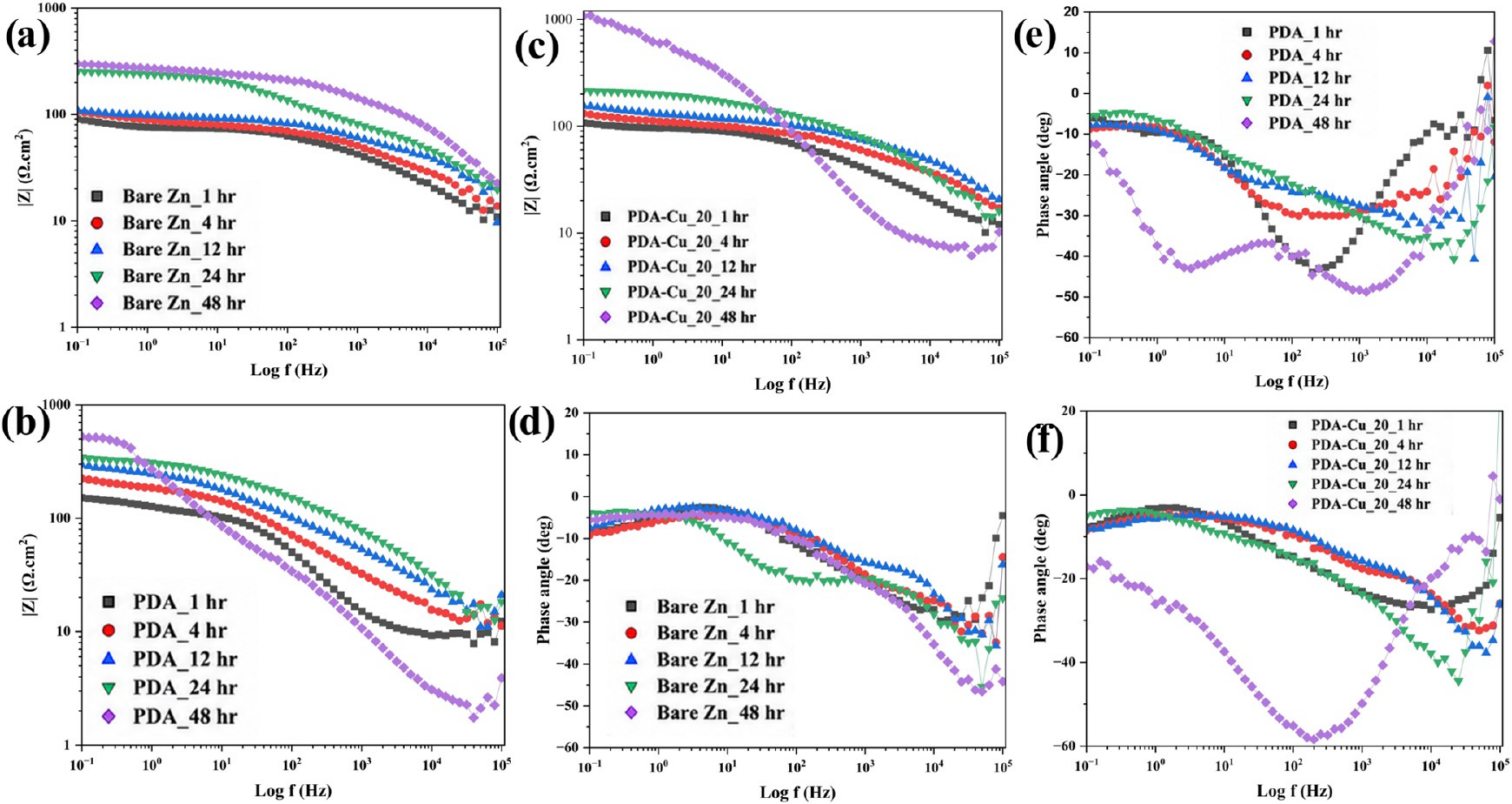
Electrochemical impedance spectroscopy. (a–c) Bode magnitude plot and (d–f) Bode phase plot of the bare Zn, PDA, PDA-Cu coatings after immersion in HBSS solution for 1, 4, 12, 24, and 48 h.

For all samples, the low-frequency impedance |*Z*| at 0.01 Hz increased with immersion time, indicating the progressive development of a corrosion product layer and stabilization of the coating system (Figure 10a–c). Among the surfaces, PDA- and PDA-Cu20-coated Zn consistently showed higher impedance values than bare Zn throughout the entire immersion period, demonstrating the beneficial barrier effect of the PDA-based coatings. After 48 h, both coatings reached |*Z*| values on the order of 10^2^–10^3^ Ω·cm^2^, which remained significantly higher than uncoated Zn, confirming improved resistance to electrolyte penetration and slower interfacial corrosion kinetics. The Bode phase plots further reinforce the protective behavior observed (Figure 10d–f). All samples demonstrated a single time constant; however, the coated surfaces exhibited higher phase-angle values in both the high- and intermediate-frequency regions compared to bare Zn. This increase in phase angle is typically indicative of a more capacitive response and a more effective barrier layer. The PDA and PDA-Cu20 coatings maintained elevated phase-angle values throughout the 1–48 h immersion period, indicating improved coating integrity, enhanced charge-transfer resistance, and reduced electrolyte access to the metal surface. In contrast, the bare Zn surface exhibited a rapid decline in phase angle over time, which reflects its lower stability and accelerated corrosion progression.

Overall, the EIS results indicate that both PDA and PDA-Cu20 coatings enhance the corrosion resistance of zinc by improving the barrier performance of the surface layer. The gradual increase in low-frequency impedance, coupled with a relatively stable phase-angle response over immersion time, suggests that these coatings effectively slow interfacial charge transfer and impede the loss of corrosive species. As a result, they offer more durable protection compared to the uncoated substrate.

The STEM studies showed that the addition of copper to the PDA polymer matrix led to noticeable changes in the particulate of the corrosion products (Figure 11). With increasing copper content, the surface gradually became covered with corrosion products after 30 days of immersion in HBSS solution. While in the uncoated samples, corrosion products were observed as irregular and nonuniform particles, the Cu-containing samples mainly showed uniform corrosion products with a rectangular morphology. In addition, the continuous accumulation of these corrosion products enclosed some of the rectangular particles between the layered corrosion products, creating a denser, thinner layer that was more resistant to the penetration of corrosive agents. Such layer increased the barrier property and more effectively protected the zinc substrate against corrosion.^35^ These findings were in full agreement with previous electrochemical results and showed that PDA-Cu coatings improve the corrosion resistance of Zn substrates in HBSS.

**Figure 11:**
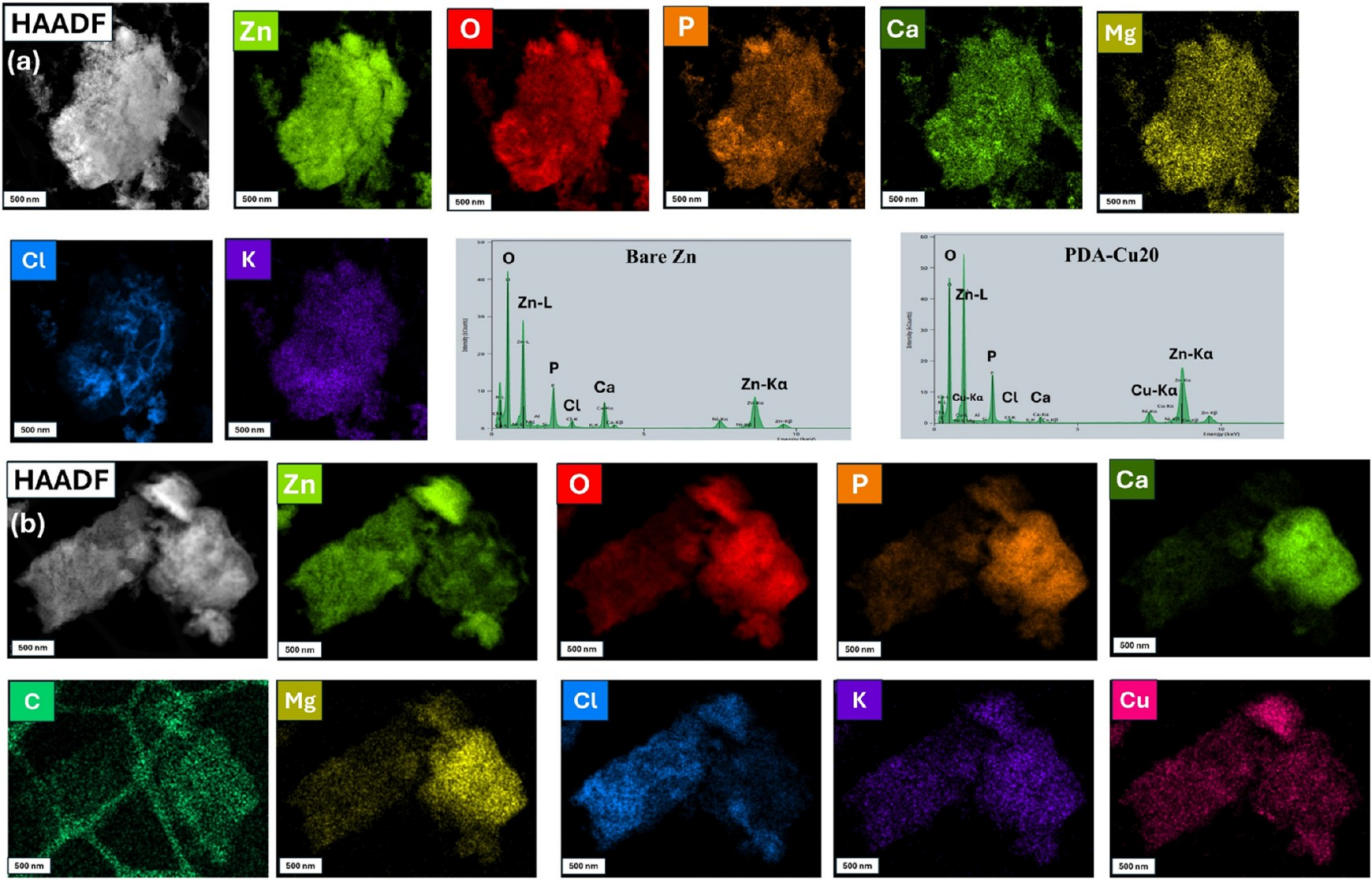
HAADF-STEM and EDS analysis (a) Bare Zn (b) PDA-Cu20 coating after 30 days of immersion in Hank’s solution (scale bar = 500 nm).

The results of EDS analysis and elemental mapping of Zn samples and PDA-Cu20 coatings after immersion test indicated a uniform distribution of elements on the surface (Figure 11). The clear presence of copper in the data showed that some of the copper complexes remained stable in the coating structure. Also, the dispersion of C, O, P, and Ca elements on the surface of the samples confirmed the presence of compounds such as carbonates and phosphates in the corrosion products. After the immersion test, a decrease in the amount of copper in the PDA-Cu coating was observed, indicating the release of some of the copper ions into the HBSB environment during the corrosion process.

The PDA-Cu coating showed better performance than the other bare Zn coatings and provided the highest corrosion resistance. This feature is attributed to the barrier effect of the coating, which prevents chloride ion (Cl^–^) penetration into the structure. Since PDA can electrostatically interact with chloride ions, this interaction reduces Cl^–^ diffusion and increases the electrochemical stability of the layer.^44,45^ As a result, the intensity of the chlorine signal in the PDA-Cu coating was significantly reduced compared to the uncoated Zn surface. Also, the high intensity of phosphorus (P) in the EDS spectra indicates the formation of phosphate corrosion products. This indicates that PDA-Cu coatings can greatly prevent pitting corrosion and metal ion dissolution in the early stages of contact with the environment by forming dense, uniform protective films, thereby increasing the surface’s durability.

Furthermore, the FTIR spectra of the corrosion products isolated from the surface of the samples showed the presence of distinct vibrational bands associated with hydrated zinc compounds and ionic species derived from the Hank medium (Figure 12a). In the range of 3200–3600 cm^–1^, a broad absorption band with a shoulder around 3650 cm^–1^ was observed, which was attributed to the stretching vibrations of the O–H bond, revealing the presence of hydroxyl groups in the Zn­(OH)_2_ compounds and physical water adsorbed on the surface.^35^ A prominent peak was observed around 1440 cm^–1^, which is related to the asymmetric stretching vibration of carbonate ions (CO_3_^2–^). This spectral feature confirms the formation of zinc hydroxycarbonate species, which are most likely formed as a result of the equilibrium between dissolved CO_2_ and bicarbonate ions (HCO_3_^–^) in Hank’s buffer solution. In addition, a band was observed near 855 cm^–1^, which corresponds to the vibrations of phosphate groups (PO_4_^3–^); in this region, the presence of HPO_4_^2–^ species is also expected to contribute to the formation of the aforementioned band.^46^ In the lower frequency region, a lattice vibration of the Zn–O bond was observed in the range of 440–450 cm^–1^, which is attributed to the formation of the ZnO phase. In samples modified with PDA–Cu coating, this band shifted and broadened in the range of 520–600 cm^–1^, indicating the formation of Cu–O bonds and the formation of Cu^2+^ chelate complexes with catechol and quinone groups present in the PDA structure. It is noteworthy that the uncoated Zn samples showed a much stronger O–H bond than the coated surfaces; this indicates the formation of larger amounts of hydrated corrosion products and, consequently, higher corrosion activity.^35^ On the contrary, in the PDA–Cu coated samples, the O–H band intensity decreased significantly, confirming the inhibitory role of the coating in reducing surface moisture absorption and inhibiting the spread of corrosion reactions.

**Figure 12:**
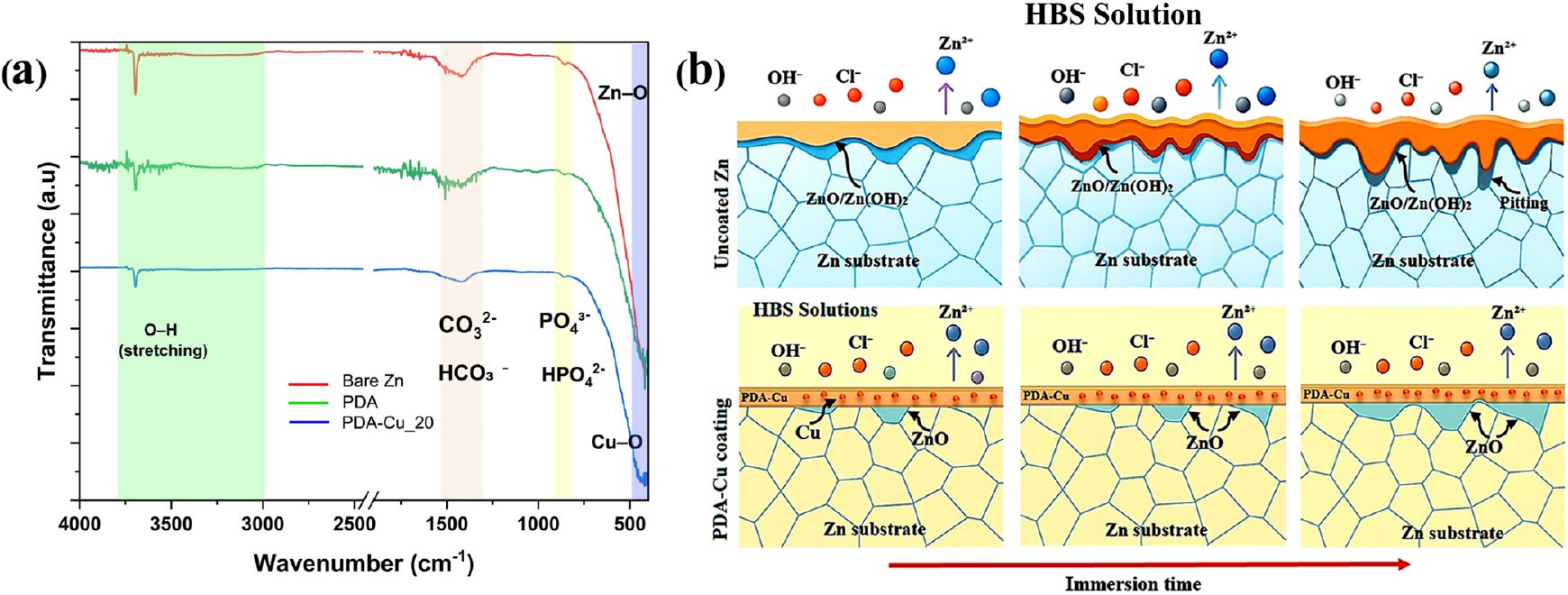
(a) FTIR analysis of bare Zn, and PDA-Cu coatings, (b) schematic illustrating the corrosion-mitigation pathway of uncoated and PDA-Cu coating.

### 3.5 Corrosion Protection Mechanism

The study of the physical and chemical properties of zinc surfaces, both in the uncoated and coated state, showed that at the beginning of the contact of the samples with the HBSB solution, the corrosion process of the oxygen absorption type begins. At this stage, the oxidation reactions on the metal surface cause an increase in the concentration of OH^–^ in the environment, and subsequently, the pH value of the solution changes toward more alkaline values. The anodic and cathodic reactions effective in this process are expressed in eqs 4 and 5. The Zn^2+^ ions resulting from the anodic reaction combine with the OH^–^ ions present in the solution and form the hydroxide compound Zn­(OH)_2_. This compound then undergoes partial conversion and is converted into ZnO, as shown in eqs 6 and 7.^35^ In fact, these developments represent the natural pathway of formation of more stable corrosion products on the zinc surface during the initial contact with the solution, which ultimately leads to the formation of a protective layer of alkaline nature.^35^

Anodic reaction

4 $$\mathrm{Zn}\to {\mathrm{Zn}}^{2+}+2e^{-}$$

Cathodic reaction

5 $$O_{2}+H_{2}O+4e^{-}\to 4{\mathrm{OH}}^{-}$$

6 $${\mathrm{Zn}}^{2+}+2{\mathrm{OH}}^{-}\to \mathrm{Zn}{\left(\mathrm{OH}\right)}_{2}$$

7 $$\mathrm{Zn}{\left(\mathrm{OH}\right)}_{2}\to \mathrm{ZnO}+H_{2}O$$

The zinc surface undergoes severe corrosion at this stage, mainly due to the autocatalytic role of Cl^–^ in the solution. This process, which usually manifests as pitting corrosion, follows the reactions specified in eqs 8 and 9.^47^ Due to the low rate of polymerization and deposition of PDA, a continuous, cohesive layer is not formed on the surface; instead, the film gradually peels off during the corrosion reaction, making it almost impossible to observe the deposited PDA coating on the surface. In a slightly alkaline environment, the zinc surface slowly oxidizes to form the ZnO compound, which is visible under steady-state conditions eq 10.^47^ However, the formation of this corrosion product occurs heterogeneous and very slowly during immersion; For this reason, the resulting layer is loose and nondense, with limited ability to prevent the penetration of chloride ions and prevent further corrosion.^18,48^ On the other hand, due to the small amount of PDA in the solution, the thickness of the formed film is not sufficient to create an effective barrier against the corrosive environment (Figure 12b). This causes the resulting coating to lack the stability and cohesion necessary to prevent surface corrosion, and as a result,^49–51^ its effectiveness in inhibiting the metal degradation process is significantly reduced.^47^

8 $$\mathrm{Zn}+H_{2}O\to {\mathrm{ZnOH}}^{+}+H^{+}+2e^{-}$$

9 $${\mathrm{ZnOH}}^{+}+{\mathrm{Cl}}^{-}\to {\mathrm{Zn}}^{2+}+{\mathrm{OH}}^{-}+{\mathrm{Cl}}^{-}$$

10 $${\mathrm{ZnOH}}^{+}+{\mathrm{OH}}^{-}\to \mathrm{ZnO}+H_{2}O$$

On the other hand, the presence of bicarbonate (HCO_3_^–^) and phosphate (HPO_4_^2–^) ions in the HBSS solution led to the formation of compounds such as Zn_5_(OH)_8_Cl_2_ and Zn_5_(CO_3_)_2_(OH)_6_ in the later stages of the corrosion process eqs 11 and 12. These reactions indicate that during surface decomposition, the interaction between ions in the solution and the primary corrosion products leads to the formation of more complex corrosion product that play an important role in the protective barrier capacity of zinc alloys.^35,52,53^

11 $$5{\mathrm{Zn}}^{2+}+2{\mathrm{Cl}}^{-}+9H_{2}O\to {\mathrm{Zn}}_{5}{\left(\mathrm{OH}\right)}_{8}{\mathrm{Cl}}_{2}\cdot H_{2}O+8H^{+}$$

12 $$5{\mathrm{Zn}}^{2+}+2H{CO}_{3}^{-}+6H_{2}O\to {\mathrm{Zn}}_{5}{\left({\mathrm{CO}}_{3}\right)}_{2}{\left(\mathrm{OH}\right)}_{6}+8H^{+}$$

Moreover, Zn_5_(OH)_8_Cl_2_ could not exist stably at too high or too low pH values; therefore, the massive release of OH^–^ was not conducive to its formation as follow:^54^

13 $$3{\mathrm{Zn}}^{2+}+2{\mathrm{HPO}}_{4}^{2-}+2{\mathrm{OH}}^{-}\to {{\mathrm{Zn}}_{3}\left({\mathrm{PO}}_{4}\right)}_{2}{\left(H_{2}O\right)}_{2}$$

When the PDA-Cu composite coating is exposed to a corrosive environment, a dense and uniform film is formed on the surface, which acts as an effective physical barrier to prevent Cl^–^ from directly contacting the zinc metal substrate. In addition, the chemical structure of polydopamine has active functional groups that can selectively adsorb chloride ions, thereby greatly reducing the possibility of reaction between the metal surface and the corrosive environment. Under such conditions, the copper metal particles in contact with the corrosive solution gradually dissolve and become soluble copper ions (Cu^2+^).^33^ These ions then react with compounds in the environment to form copper oxides, including CuO and Cu_2_O, on the surface. Overall, the developed PDA and PDA-Cu coating slows down the degradation of the Zn substrate and reduces the degradation rate.

## 4 Conclusion

In this study, an ultrathin, dense polydopamine-based coating containing divalent copper ions (Cu^2+^) was developed for biodegradable Zn stents to enhance nitric oxide generation and improve corrosion resistance. The presence of copper ions in the polydopamine structure accelerated the formation of the coating layer, regulated surface chemistry, maintained coating integrity, reduced the rate of zinc dissolution, and steadily released copper ions. On the other hand, the catalytic activity of Cu^2+^ in reactions with nitric oxide donors (NO donors) led to NO production, confirming the coating’s effective physiological function in the biological environment. The results of electrochemical tests showed that the presence of the coating directly changed the surface behavior and significantly increased the corrosion resistance. The corrosion rate decreased from 0.80 to 0.40 mm/year, which can be attributed to the synergistic effect of copper ions in the coating layer. Microscopic images showed that the coating was formed uniformly, smoothly and without porosity. This dense structure increased the corrosion resistance compared to the uncoated zinc surface. Overall, the PDA-Cu composite coating is introduced as a compelling, multipurpose protective film that delivers sustained Cu ion release, NO generation, and high anticorrosion performance, with a high potential for use in the design and development of biodegradable stents.

## Supplementary Material



## Data Availability

Data sets supporting the findings of this study are available from the corresponding author upon reasonable request.

## Notes

Schematic illustration of the synthesis and coating process for PDA and PDA–Cu coated Zn samples, including Cu^2+^ incorporation into the PDA matrix, together with supplementary morphological and compositional characterization (PDF)
