# Supplementary material for: Polydopamine-Cu(II) Ions Functional Coatings on Zinc Wire: Surface Characterization and Degradation Behavior
Source: ACS Mater Au. 2026 May 20;6(4):896–911. doi: 10.1021/acsmaterialsau.6c00093 (PMC13352281; doi:10.1021/acsmaterialsau.6c00093)
Supplement: Supplementary file 1 [file mg6c00093_si_001.pdf]

Supporting Information

Polydopamine-Cu (II) Ions Functional Coatings on Zinc Wire: Surface Characterization and Degradation Behavior

Md Tanvir Hossain[1], Hamid Reza Bakhsheshi-Rad[1,2], Bashir Ahamed[1], Erico Freitas[1], Bruce P. Lee[3], Jeremy Goldman[3], Jaroslaw W. Drelich[1,*]

[1]Department of Materials Science and Engineering, Michigan Technological University, Houghton, MI, 49931, USA
[2] Department of Materials Engineering, Na.C., Islamic Azad University, Najafabad, Iran
[3]Department of Biomedical Engineering, Michigan Technological University, Houghton, MI, 49931, USA

*Corresponding author's email: jwdrelic@mtu.edu

Zn wires and disk-shaped Zn substrates were polished, ultrasonically cleaned, and coated with PDA and PDA–Cu layers through a mussel-inspired immersion-assisted polymerization process in Tris-HCl buffer (pH 8.5). $CuCl_2$ (5–20 mg/L) was incorporated into the dopamine solution to fabricate PDA–Cu coatings. After 8 h immersion under gentle stirring, the coated samples were rinsed with ethanol and air-dried prior to characterization and corrosion testing. The surface morphology, coating uniformity, elemental distribution, and cross-sectional structure of bare Zn and PDA/PDA–Cu-coated samples were characterized using field-emission scanning electron microscopy coupled with FE-SEM/EDS. Coating thickness and PDA nanosphere size distribution were quantified from SEM images using ImageJ software. In addition, STEM and HAADF-STEM analyses were performed to evaluate the nanoscale morphology and Cu distribution within the PDA matrix, while EDS mapping was used to confirm the elemental composition and uniformity of Cu incorporation (Fig. S1).

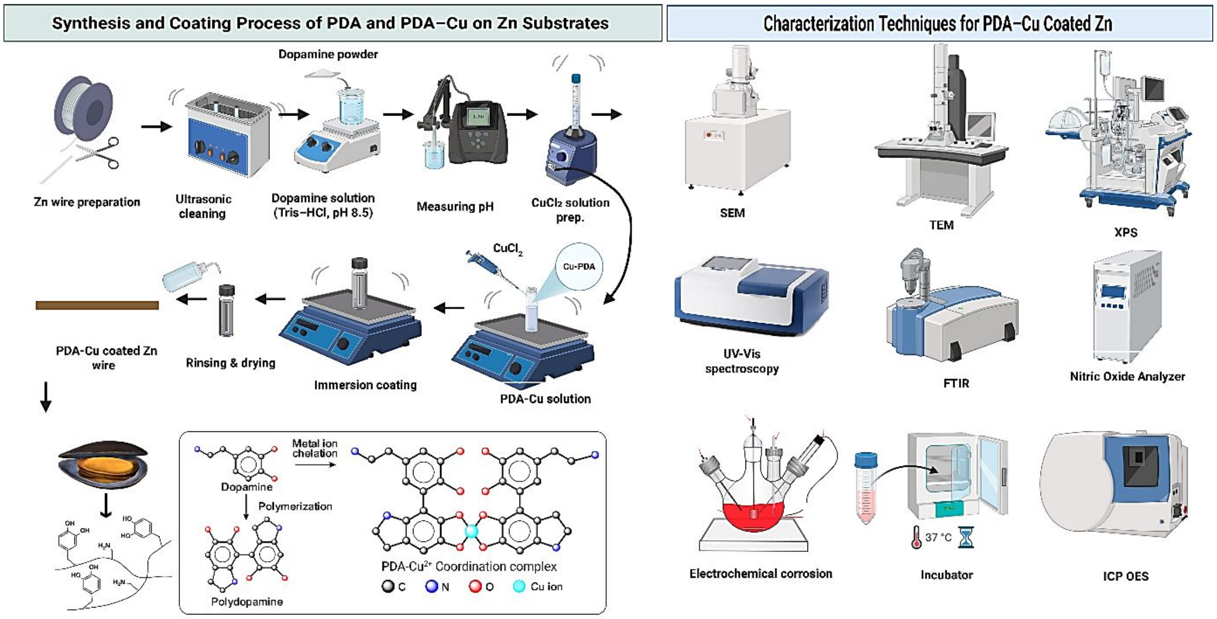

Figure S1. Schematic illustration of the synthesis and coating process, along with the corresponding characterization techniques, showing the incorporation of Cu^{2+} ions to form coordination complexes within the PDA matrix.
